# Disease risk analysis for schizophrenia patients by an automatic AHP framework

**DOI:** 10.1186/s12911-022-01749-1

**Published:** 2022-01-11

**Authors:** Wenyan Tan, Heng Weng, Haicheng Lin, Aihua Ou, Zehui He, Fujun Jia

**Affiliations:** 1 Guangdong Mental Health Center, Guangdong Provincial People’s Hospital, Guangdong Academy of Medical Sciences, Guangzhou, People’s Republic of China; 2 State Key Laboratory of Dampness Syndrome of Chinese Medicine, The Second Affiliated Hospital of Guangzhou University of Chinese Medicine, Guangzhou, People’s Republic of China

**Keywords:** AutoAHP, Risk analysis for severe psychosis, Intelligent information processing, Disease risk prediction

## Abstract

**Background:**

Based on more than 15 million follow-up records of 404,426 patients from Guangdong Mental Health Center over the past 10 years, this study aims to propose a disease risk analysis and prediction model to support chronic disease management and clinical research for schizophrenia patients.

**Methods:**

Based on a mental health information and intelligent data processing platform, we design an automatic AHP framework called AutoAHP to analyze and predict the disease risks of schizophrenia patients. Through automatic extraction, transformation and integration of follow-up data in the real world such as demography, treatment, and the disease course, a chronic database of patient status is established. In combination with age-period-cohort, logistic regression and Cox models, we apply the AutoAHP to assess disease risk and implement risk prediction in practice.

**Results:**

A list of essential factors for risk prediction are identified, including annual changes in mental health policy, public support, regional difference, patient gender, compliance, and social function. After the verification of 1,222,038 complete disease course and treatment records of 256,050 patients, the AutoAHP framework achieves a precision of 0.923, a recall of 0.924, and a F1 of 0.923. The model is demonstrated to be superior to general models and has better performance in risk prediction.

**Conclusions:**

Aiming at the risk assessment of patients with schizophrenia which is influenced by factors, such as time, region and complication, the AutoAHP framework is able to be applied as a model in combination with logistic regression and Cox models to support clinical analysis of disease risk related factors and assist decision-making in chronic disease management.

**Supplementary Information:**

The online version contains supplementary material available at 10.1186/s12911-022-01749-1.

## Background

According to the statistical disease registration platform of Guangdong Mental Health Center, there are more than 400,000 schizophrenic patients in Guangdong province, and shows an increasing trend [1]. Pathological impulsivity and risk-taking are common in patients, and have clinical repercussions, including novelty seeking, response disinhibition, aggression, and substance abuse, which has severe consequences for patients themselves, their families and the society [2, 3]. It is of great significance to utilize intelligent technologies to predict the occurrences of risk events, strengthen disease prevention and control, reduce the incidence of risk events, and assist decision-making [4, 5].

Nationwide register-based data has been used to conduct a prospective population-based cohort study of patients with schizophrenia, as previously described. It is meaningful to find out whether there are any clinically differences among specific antipsychotic medications or routes of administration regarding the risk of psychiatric re-hospitalization, suicide or other treatment failures [6, 7]. Since the risk events of patients is regarded as robust evidence for decision-making, data should be generated in a methodologically sound, structured, and transparent way [8]. The analytic hierarchy process (AHP) organically combines qualitative and quantitative methods and decomposes a decision into a multi-level hierarchical structure. In this way, the thinking processes of decision makers are systematized and simplified [9, 10].

AHP and relevant comprehensive decision support frameworks have been developed for factor analysis, safety management, and quality evaluation etc. [11–14]. However, the follow-up data of mental illness in communities is characterized by long-time cycle, non-linearity and complex relationship among variables. The existing methods of manual AHP construction are time-consuming and may laborious. In addition, since the prediction of illness risk is influenced by some subjective factors, it is difficult to effectively combine objective and subjective factors together.

To that end, on the basis of the investigation of existing researches, this study develops an automatic AHP framework called AutoAHP for disease risk analysis and prediction from the data of schizophrenia patients [15–19]. Specifically, this paper has conducted the multidimensional analysis including: (1) automatic extracting and marking of risk events, including ‘behavior that endangers society’, ‘hospitalization/referral’, ‘loss of self-knowledge’, and ‘suicide/death’; (2) screening and normalizing relevant variables including ‘demography’, ‘treatment, and ‘disease course’; (3) utilizing multi-factor analysis, mixed-effect model of APC (age, period and course of disease) and other methods to assist experts to construct the criterion layer of AHP; (4) establishing, verifying and applying the disease risk analysis and prediction model for schizophrenia patients. The data is from a mental health information and big data intelligent processing platform from the Guangdong mental health center. The platform has collected 15 million follow-up records of 404,426 patients over 10 years [20].

## Methods

Based on the practice guideline for the treatment of patients with schizophrenia [19], the monitoring data of patients with schizophrenia from January 2010 to November 2019 is derived from Mental Health Information and Big Data Intelligent Processing Platform [20], involving 404,426 patients and a total of 15 million follow-up records. The data is multi-source and heterogeneous, including patient disease registration files, follow-up records, medication records, physical examination reports, hospitalization records, etc. All follow-up data are grouped by patients and treatment plans. Referring to scalable and accurate deep learning with electronic health records [21] and data models for follow-up management of schizophrenia, 1.2 million structured data covering 256,050 patients are formed. The data contains 48 variables, involving age, disease course, disease state, treatment interventions, efficacy and adverse reactions, and outcome events. In order to train and verify the model, the data set is divided into a training set and a test set in a ratio of 2:1.

In this study, the data is subjected to age period cohort analysis, variable screening and discretization. An automatic AHP framework AutoAHP is designed for disease risk scoring and outcome prediction of schizophrenia patients. More details are introduced in the following sub-sections.

### Factor analysis

Follow-up data over a 10-year period is influenced by society, economy, health conditions and policies. APC models [22] are extensively used in actuarial sciences, demography, epidemiology and social sciences. They have an identification problem in that the predictor is defined by time effects for the APC (age, period and cohort) factor. However, these time effects cannot be fully recovered from the predictor [23, 24]. In this study, APC analysis method is used to estimate this effect, and a cohort is generated by setting age and period as fixed effect and the cohort as random effect.

**Fig. 1:**
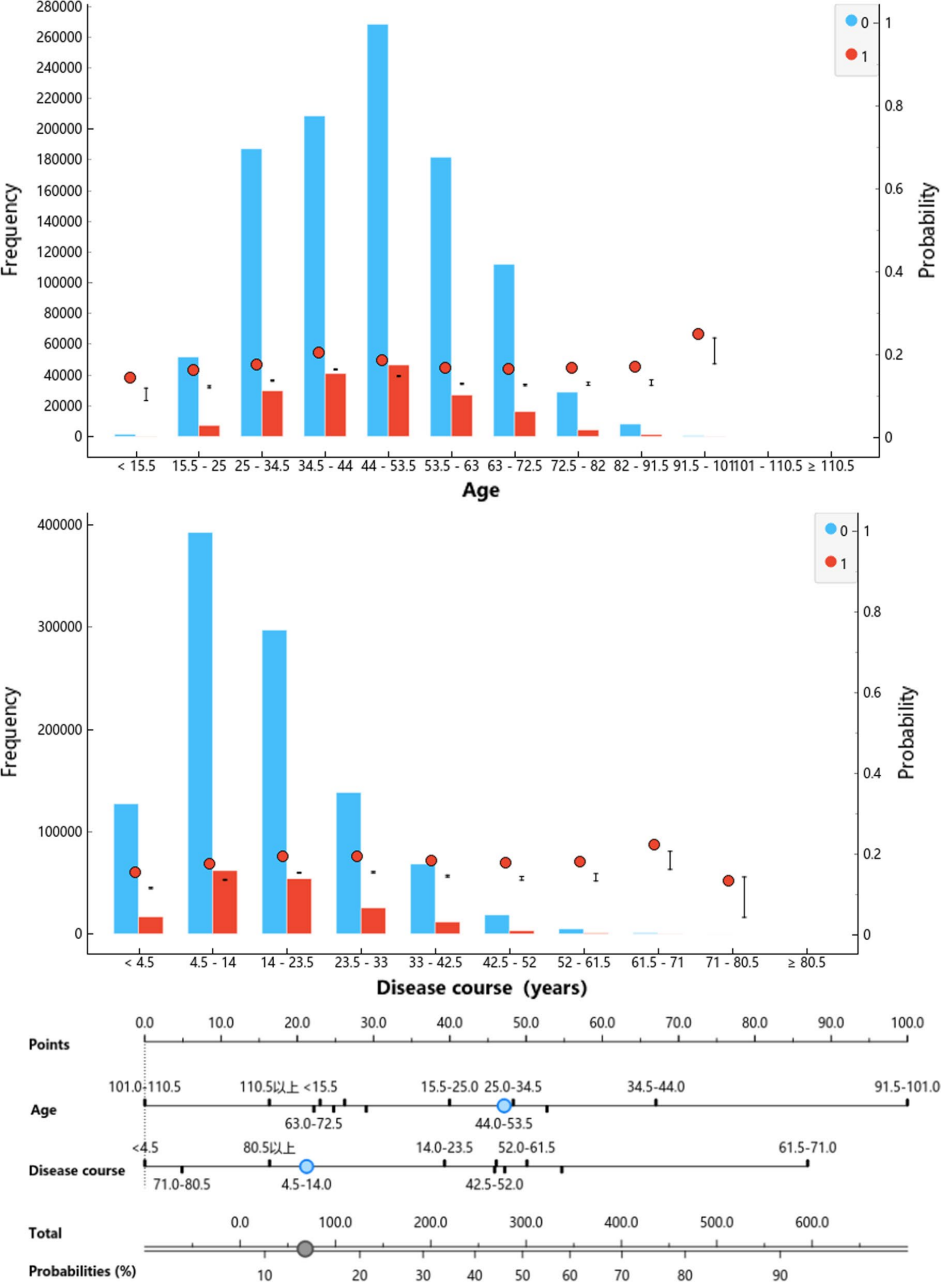
The visualization of the discretization method, where the red column indicates risk events, blue indicates no risk events, area indicates a population density distribution, and dotted line indicates a population ratio

The relationship between continuous variables, such as age, duration of disease, and risk is nonlinear. It is necessary to discretize continuous variables into categorical variables and estimate the average impact of risk variables on different intervals. The framework discretizes continuous variables based on the range and confidence of probability fluctuations and nomogram scores. The discretization method is encapsulated into an interactive visualization, as shown in the Fig. 1.

**Fig. 2:**
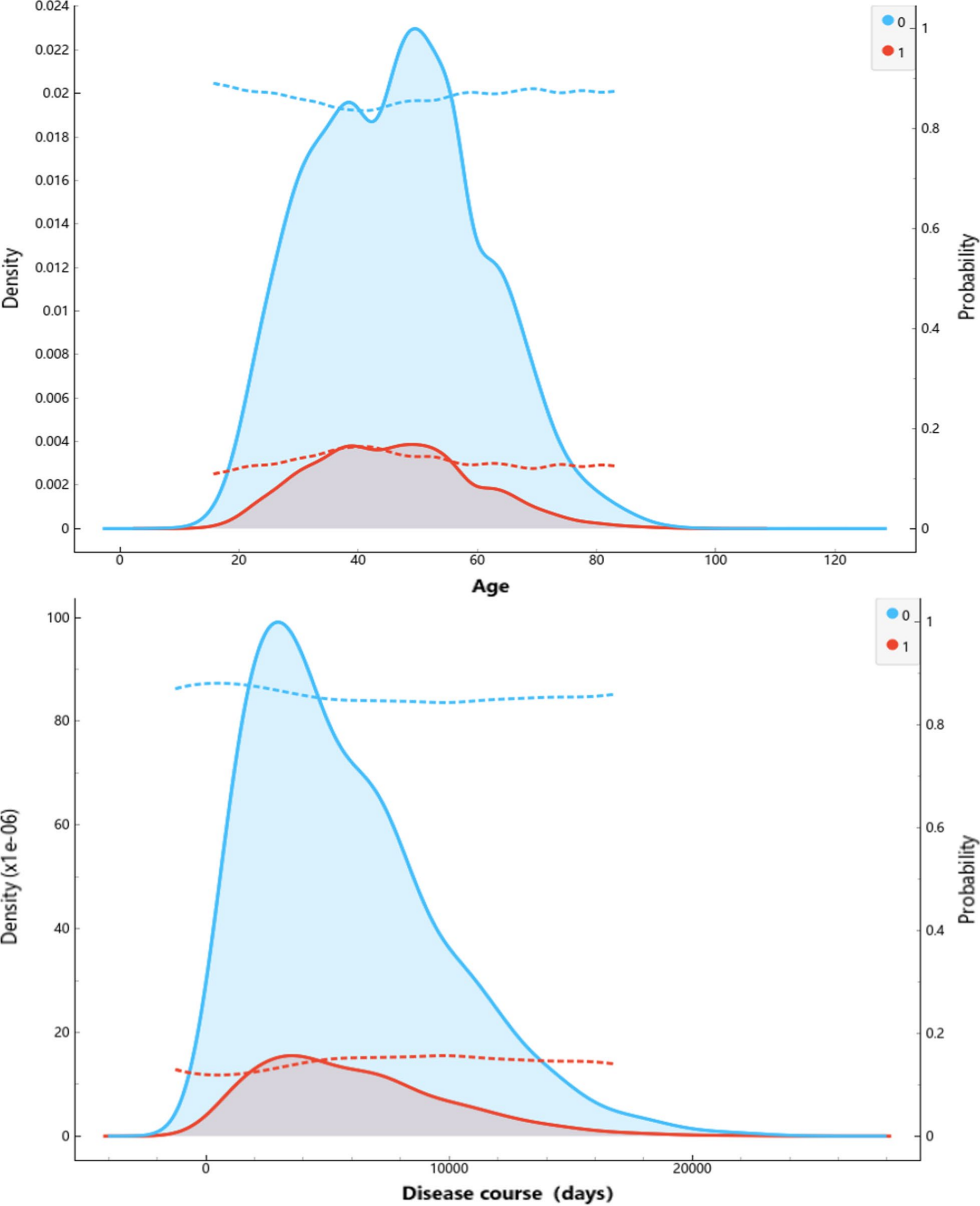
The distributions of age and disease course, where the red line indicates risk event, blue line indicates no risk events

The visualization describes the variables included in the patient state time series database, and supports experts to define rules according to the statistical results so as to establish the workflow of automatic normalization processing of the original variables. The interval information of the discretized variables is shown in Fig. 2 and Additional file 1: Table S1.

Feature selection is an essential part of feature engineering, which aims to screen out important features or eliminate irrelevant features. In this study, 23 variables are selected from 48 variables by incorporating machine learning-based feature rankings and the opinions of clinical experts. The machine learning-based rankings are the average of using indicators consisting of Information Gain (IG), Gain ratio, Gini [25], Chi-square coefficient [26], Relief F and Fast Binary Feature Selection (FCBF) [27].

### The AutoAHP framework

Through the analysis, we define the features layer by layer for analysis. The first layer includes adverse reaction, admission time, compliance, risk event, region, annual policy, age, gender, education, disability, and treatment. According to different attributes, they are decomposed into several levels from top to bottom in the way as described in [8]. The factors at the same level are subordinated to the factors at upper level or have influence on the factors at upper level, while at the same time dominating the factors at lower level or influenced by them.

**Fig. 3:**
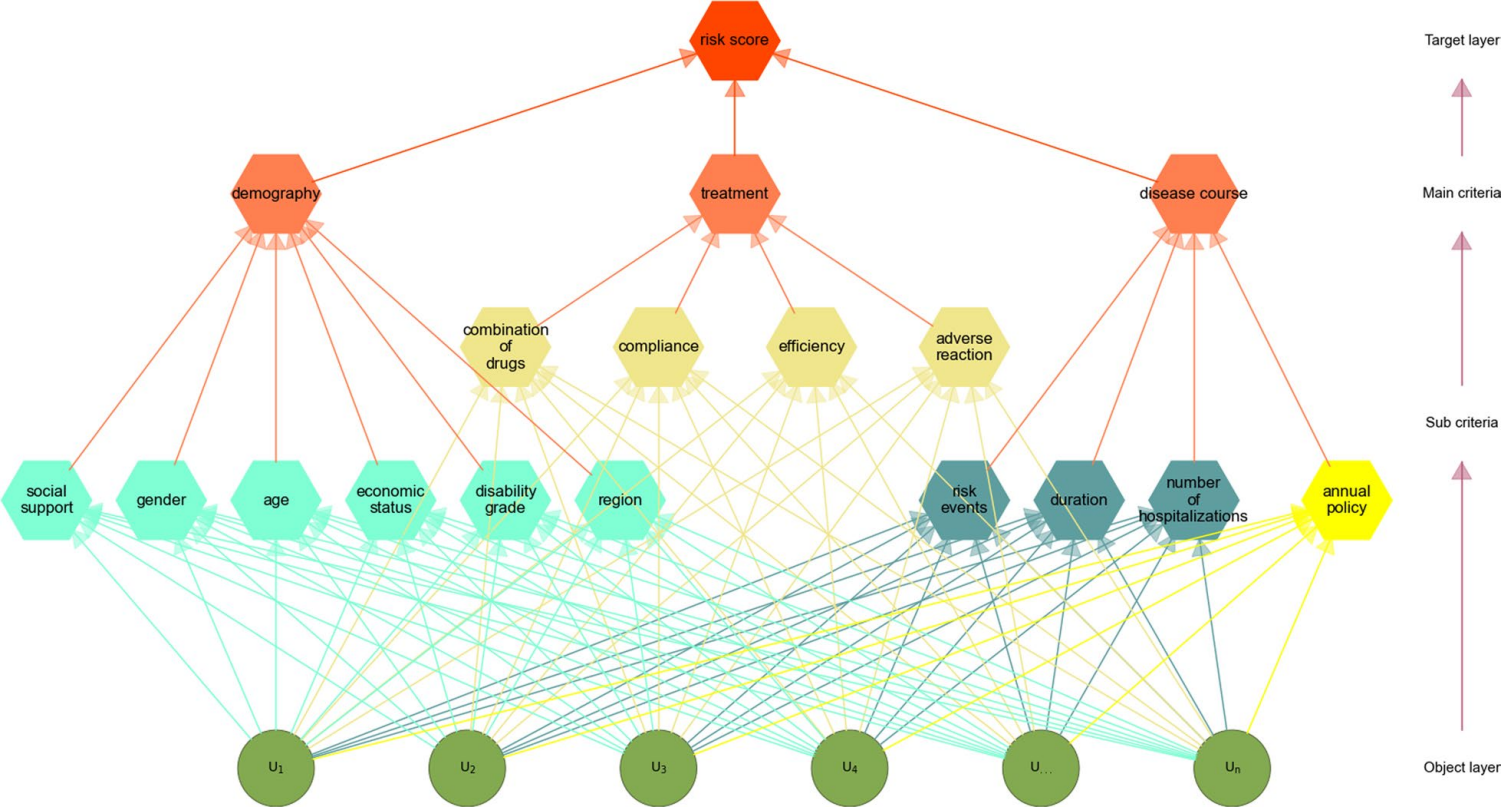
The overview of our AutoAHP framework for factor analysis

The overview of our proposed AutoAHP framework is shown in Figure 3. The top layer is the target layer with only one factor “risk score”. The lowest layer is the scheme or object layer, such as “multidimensional patient rating scale”. There may be one or more levels in the middle, usually criteria or index levels. In this study, we further decompose the criteria into sub-criteria layers. Based on the attribute analysis of the set of factors predicted by disease risk analysis of schizophrenia patients, all the risk factors are decomposed into three main criteria and several sub criteria [9], as shown in Table 1.

**Table 1:** The categories of criteria and sub-criteria of all the risk factors

Main criteria	Sub-criteria
Demography	Region, age, gender, education, disability grade, social support, economic status
Treatment	Adverse reaction, compliance, treatment, combination of drugs
Disease course	Number of hospitalizations, risk events, annual policy, duration

### Construction of pair-wise comparison matrix

Starting from the second level of the hierarchical AutoAHP model, the comparison matrix is constructed from the pair-wise comparison scale and 1-9 comparison scale to the lowest level of the factors belonging to or affecting each factor of the upper level.

The value of ${A}_{\text{ij}}$ in the pair-wise comparison matrix comes from Saaty’s scheme and is assigned according to the following scale. The value of ${A}_{\text{ij}}$ is between 1–9 and its reciprocal:

If ${A}_{\text{ij}}$ is 1, element $i$ and element $j$ are equally important to the factors at the previous level;

If ${A}_{\text{ij}}$ is 3, $i$ is moderately more important than $j$;

If ${A}_{\text{ij}}$ is 5, $i$ is more essential than $j$;

If ${A}_{\text{ij}}$ is 7, $i$ is strongly important than $j$;

If ${A}_{\text{ij}}$ is 9, $i$ is extremely important than $j$;

If ${A}_{\text{ij}}$ is $2n$, *n* equals to 1, 2, 3, or 4, the importance of $i$ and $j$ is between ${A}_{\text{ij}}=2n-1$ and ${A}_{\text{ij}}=2n+1$.

When comparing the importance of element $i$ with that of element $j$ in relation to a factor in the previous layer, the relative weight of ${a}_{ij}$ is quantified. If *n* elements are assumed to participate in the comparison, the pair-wise comparison matrix is$A=({a}_{ij}{)}_{n\times n}$. Theoretically, if $A$ is a perfectly consistent pairwise comparison matrix, there should be ${a}_{ij}{a}_{ik}={a}_{ik},1\le i,j,k\le n$. However, the flexibility of the comparison matrix lies in that the ${A}_{ij}$ can be fine-tuned to improve model performance, which may lead to inconsistency in the comparison matrix. Therefore, the consistency of the pairwise comparison matrix *A* needs to be further tested with the following steps.

(1) The $CI$ (consistency index) is calculated for evaluating the pair-wise comparison matrix by using Eq. (1).

1 $$CI=\frac{{\lambda }_{max}(A)-n}{n-1}$$

where $n$ is the dimension of the matrix and ${\lambda }_{max}$ is the maximal eigenvalue of the matrix.

(2) The random consistency ratio $CR$ (consistency ratio) for comparison matrix is calculated by using the Eq. (2).

2 $$CR=\frac{CI}{RI}$$

$RI$ refers to random index, which is only related to matrix order $n$ (usually no more than 9). The standard $RI$ is for checking the consistency of pair-wise comparison matrix $A$ according to relevant data. If $CR$ is less than 10%, the matrix is considered to have an acceptable consistency. Otherwise, the pair-wise comparison matrix $A$ is adjusted until the satisfactory consistency is achieved.

### Computation of weighted vectors

For each pair-wise comparison matrix, the maximum eigenvalues and corresponding eigenvectors are calculated. The consistency tests are performed with the $CI$, $RI$ and $CR$. If the test passed, the eigenvectors are normalized as weight vectors. Otherwise, the pair-wise comparison matrix needs to be reconstructed. After normalization, the eigenvectors are computed by using Eq. (3).

3 $${\lambda }_{max}=\frac{\sum (AW{)}_{i}}{n{W}_{i}}$$

For the initialization of criteria layer weights, a procedure has been designed. Taking the second layer construction as an example, the procedure consists of: (1) initializing the weights of factor nodes; (2) defining the weight set of nodes *Fw* by artificial or machine learning ways; (3) generating the initial pair-wise comparison matrix *A* according to *Fw* and adjust *A*; (4) checking the consistency of *A* after adjusting; 5) calculating the standard weights of element nodes $\left\{{a}_{1},{a}_{2},\dots ,{a}_{n}\right\}$.

Based on the Auto-AHP framework, the criterion layer and pair-wise matrix are automatically defined, in which the combination of manual and machine learning ways is the key to optimization. Eventually, the AutoAHP framework supports decision makers in following ways: (1) Analysis of age-period-course factors of long-term epidemiological big data; (2) Machine learning correction of standardized conversion of nonlinear continuous variables and classified variables, weight allocation among multiple factors, and other aspects; (3) The consistency ensurence of pair-wise comparison matrix during manual-machine combination correction.

The combination of weight vectors of the lowest level to the target is calculated, and the combination consistency test is carried out. If passed the test, the decision can be made according to the result represented by the combination weight vectors. Otherwise, the model needs to be reconsidered or the matching comparison matrix with large consistency ratio needs to be reconstructed. Manual fine-tuning is conducted through scoring feedback and decision results.

Based on the propensity score of AutoAHP framework, a synthetic risk prediction score of hazardous events is condensed by using several known independent variables. On the one hand, it can be used in disease risk prediction tasks. On the other hand, it can also be used to deal with problems caused by unbalanced data. Study subjects are selected from the experimental group and the control group to form a new one. Then the effects of intervention factors are compared between the two groups, such as the comparison of drug use, compliance and so on between different groups.

### Baseline methods

The risk assessment of schizophrenia is treated as a classification problem. A number of commonly used machine learning classification methods are used as baselines. Statistical analysis and baseline methods are implemented in Scikit-learn Python. The methods and related hyper-parameters setting are shown in Table 2. All methods are evaluated by five widely used classification measures: area under the curve (AUC), accuracy, precision, recall and F1-measure.

**Table 2:** Hyper-parameters of baseline methods

Methods	Hyper-parameters
Random Forest	num of trees: 1000, num of attr consider at each split: 5
Neural Network	Neurons of hidden layers: 100, activation: Relu, solver: Adam, regularization, learning rate: 0.001, iters: 200
Logistic Regression	regularization type: ridge(L2), strength: *C* = 1
SGD	Loss function: logistic regression, regularization method: Elastic Net, $\epsilon$: 0.1, iters:1000
kNN	*K*: 9, metric: Euclidean, weight: Uniform
SVM	RBF, Kernel:$exp(-g|x-y{|}^{2})$, *C*: 1.00,: 0.1, iteration limit: 100

## Results

**Fig. 4:**
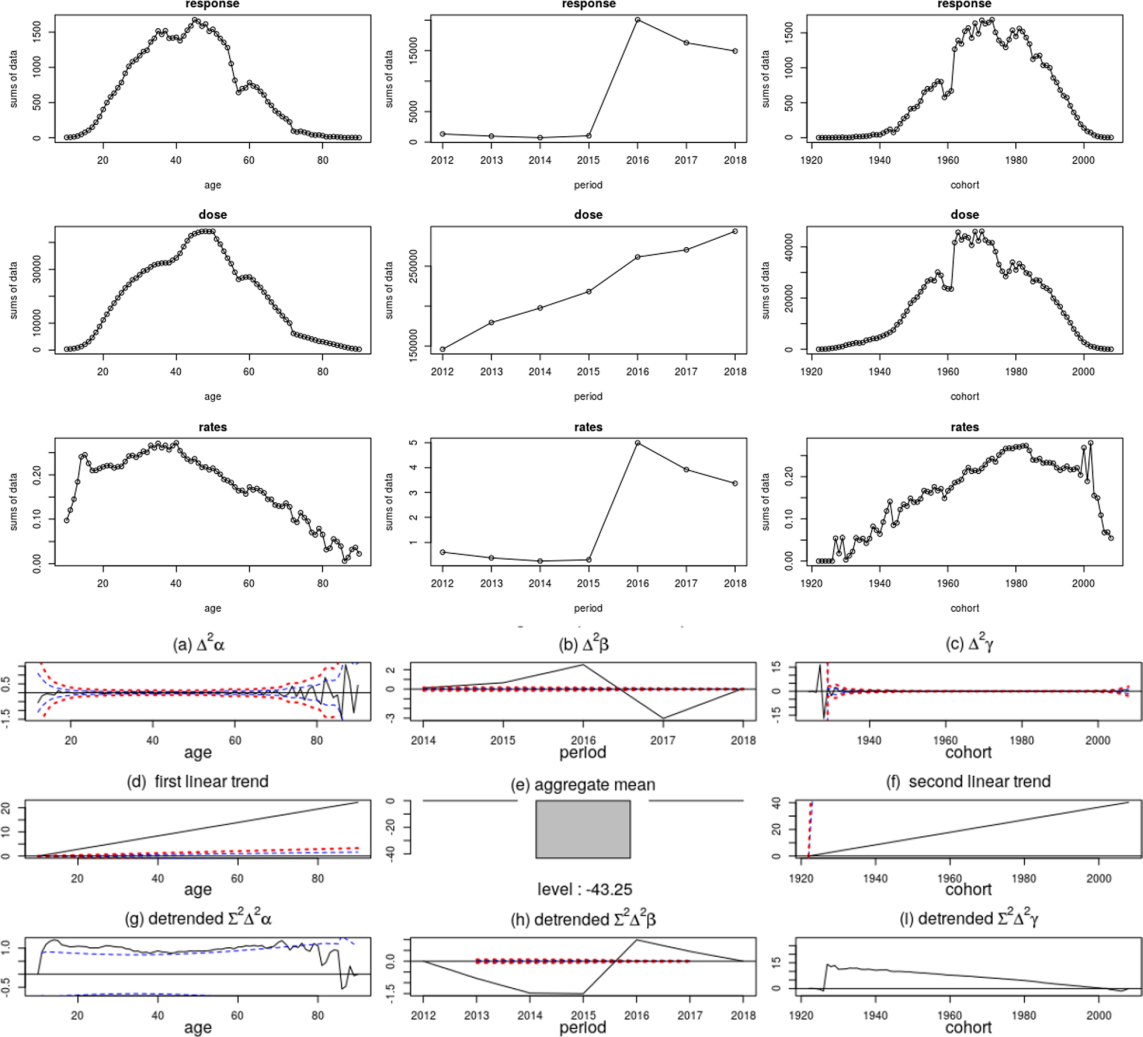
Risk events data by age cohort period index and APC canonical parameters as well as representation of follow-up data of schizophrenia patients

Through analysis of age-period-cohort factors of long-cycle epidemiological data, the response and rates increased over one period is reported in Fig. 4. The result indicates that there are significant events related to chronic disease management in schizophrenia patients in Guangdong Province. Furthermore, we implement the AutoAHP framework with the period parameters in mixed-effect model of APC (age, period and course of disease) to eliminate the influence of annual policy change (*n*) on the occurrence of risk events and hospitalization rate (4–6).

In combination with clinical expert experience and machine-learning feature selection, information gain, chi-square and other methods are carried out for variables in the follow-up data for the risk prediction of risk events. The set of factors related to the risk prediction of dangerous events is screened by examining each variable. Table 3 shows the final features and related indicators as detailed in Additional file 1: Table S2.

**Table 3:** The final features and related indicators

	Info. gain	Gain ratio	Gini	${X}^{2}$	ReliefF	FCBF
Referral times*	0.034	0.024	0.021	2881.741	0.056	0
Drug combination	0.013	0.003	0.008	1037.934	0.112	0
Compliance	0.011	0.026	nan	892.802	0.02	N/A
Adverse reactions times*	0.003	0.005	0.002	339.393	0.001	0
Suggest Referral	0.036	0.07	0.019	168.791	0.038	0.05
Diagnostic type	0.004	0.005	0.003	141.892	0.052	0
Social function	0.006	0.008	0.004	136.586	0.024	0.007
Poverty	0.003	0.003	0.002	129.539	− 0.002	0
Targeted poverty alleviation	0.003	0.003	0.002	129.539	− 0.002	0
Duration days*	0.002	0.001	0.001	85.161	0.02	0
Family guardianship subsidy	0.006	0.01	0.004	60.961	0.006	0
Auxiliary drug combination	0.003	0.001	0.002	59.541	− 0.005	0
Disability rating	0.002	0.001	0.001	38.923	0.128	0
Hospitalization times	0.001	0.001	0.001	36.31	0.027	0
Region	0.063	0.022	0.042	21.243	0.142	0.034
Gender	0	0	0	16.812	0.004	0
Course of disease rating	0.001	0.001	0.001	13.776	0.048	0
Region	0.001	0.002	0	10.812	0.032	0
Drug combination number*	0	0	0	9.131	0.011	0
Age rating	0.001	0.001	0	5.367	0.036	0
Economic status	0	0	nan	2.868	0.068	N/A

*denotes the variables that are discretized

On the basis of the linear transformation, we further introduce the Cox regression analysis method to calculate and compare the risk weights of occurrence of dangerous events among variables under the premise of duration. The results of Cox analysis is shown in Table 4. In addition to the level of education, all the variables on the correlation with risk events have statistical significance, including compliance (1 refers to good status), social support, gender (0 is male and 1 is female) and social function, in which the regional difference is significant. We take HR (95% CI) coefficient as the element nodes of the AutoAHP model to build the basis for the pair-wise comparison matrix.

**Table 4:** Parameter tests of Cox regression

	Coefficients [95% CI]	z
Adverse reactions times	0.01 [0.01, 0.01]	4.43*
Drug combination	0.16 [0.12, 0.19]	8.44*
Age rating	0.09 [0.05, 0.13]	4.42*
Diagnostic type	0.61 [0.54, 0.68]	17.12*
Duration days	0.05 [0.01, 0.08]	2.55*
Drug combination	− 0.06 [− 0.08, − 0.03]	− 4.03*
Gender: female	− 0.37 [− 0.41, − 0.33]	− 17.98*
Disability rating	− 0.06 [− 0.09, − 0.03]	− 4.56*
Social function	0.25 [0.21, 0.28]	13.85*
Compliance	0.48 [0.44, 0.52]	26.31*
Targeted poverty alleviation	0.31 [0.27, 0.35]	15.12*
Family guardianship subsidy	0.39 [0.32, 0.45]	12.29*

^*^$$P<0.001$$

All the evaluation measures are used to assess the performance of the AutoAHP framework and the baselines. The results, as shown in Table 5, show that the AutoAHP framework has achieved an AUC of 0.954, an accuracy of 0.924, a precision of 0.923, a recall of 0.924, and a F1 score of 0.923, being the best among all the methods. Random Forest method obtains the second top performance with a F1 score of 0.919, while SVM acquires the worst performance with a F1 score of 0.602 only.

**Table 5:** Performance comparison of the AutoAHP framework against baseline methods

Method	AUC	Accuracy	Precision	Recall	F1
SVM	0.480	0.589	0.619	0.589	0.602
kNN	0.537	0.727	0.639	0.727	0.657
SGD	0.564	0.722	0.686	0.722	0.697
Logistic Regression	0.722	0.758	0.719	0.758	0.700
Naive Bayes	0.712	0.763	0.732	0.763	0.728
Neural Network	0.900	0.881	0.878	0.881	0.879
Random Forest	0.945	0.921	0.919	0.921	0.919
AutoAHP	0.954	0.924	0.923	0.924	0.923

## Discussion

The analytic hierarchy process (AHP) organically combines qualitative and quantitative methods and decomposes a decision into a multi-level hierarchical structure. In this way, the processes for decision makers are systematized and simplified. However, it still has some limitations on solving long-term, cross-regional, multi-source heterogeneous big data and nonlinear medical problems.

The design of the AutoAHP framework for the disease risk analysis and prediction of Schizophrenia patients is based on more than 15 million follow-up records of 404,426 patients in Guangdong mental health center over recent 10 years. We conduct linear transformation and quantization of these records to alter the inadequacy of machine learning through the internal causal logic of clinical experience and regional policy so as to improve the prediction performance of the model. The AutoAHP framework introduces survival models to predict disease risks, so as to solve two problems. Firstly, duration issue can be studied. Secondly, risk factors can be thoroughly interpreted. Meanwhile, the mixed-effect model of APC (age, period and course of disease) is introduced for age-time-course analysis, which reveals the impact of policy and program changes from the perspective of long-term big data epidemiology.

The results of APC model in Fig. 4 have shown that response and rates increased over one period. In this period, the government, health management, civil affairs, public safety and other departments jointly promulgate the “Mental Health Management Policy”. Relevant policies have strengthened the definition and control of risk behaviors of patients with schizophrenia, and given more preference in medical treatment, health, and insurance, so that more patients can be hospitalized.

The results of cox regression are similar to previous studies. It demonstrates that people in low-income families who receive medical aid are more likely to have dangerous events [28–30]. Previous study indicates a significant association between suicide and disability when controlling various potential confounders, including both age and income level [31]. Physical or mental limitations due to disability are also closely linked to suicide death [32, 33].

In Guangdong Province, there is still a shortage of psychiatrists. The management level of chronic disease still needs to be improved, and subjective risk assessment may be biased. Based on the AutoAHP framework, we have analyzed some related factors, such as patient age, gender, geography, culture, economy, disease course, medication generation, dosage forms, and auxiliary drug, to assist risk prediction and even to improve the efficiency of supervision. Moreover, this is conducive to finding potential patients in risk timely, to strengthening case management, community service, and to enhancing the active intervention of patients and their families.

**Fig. 5:**
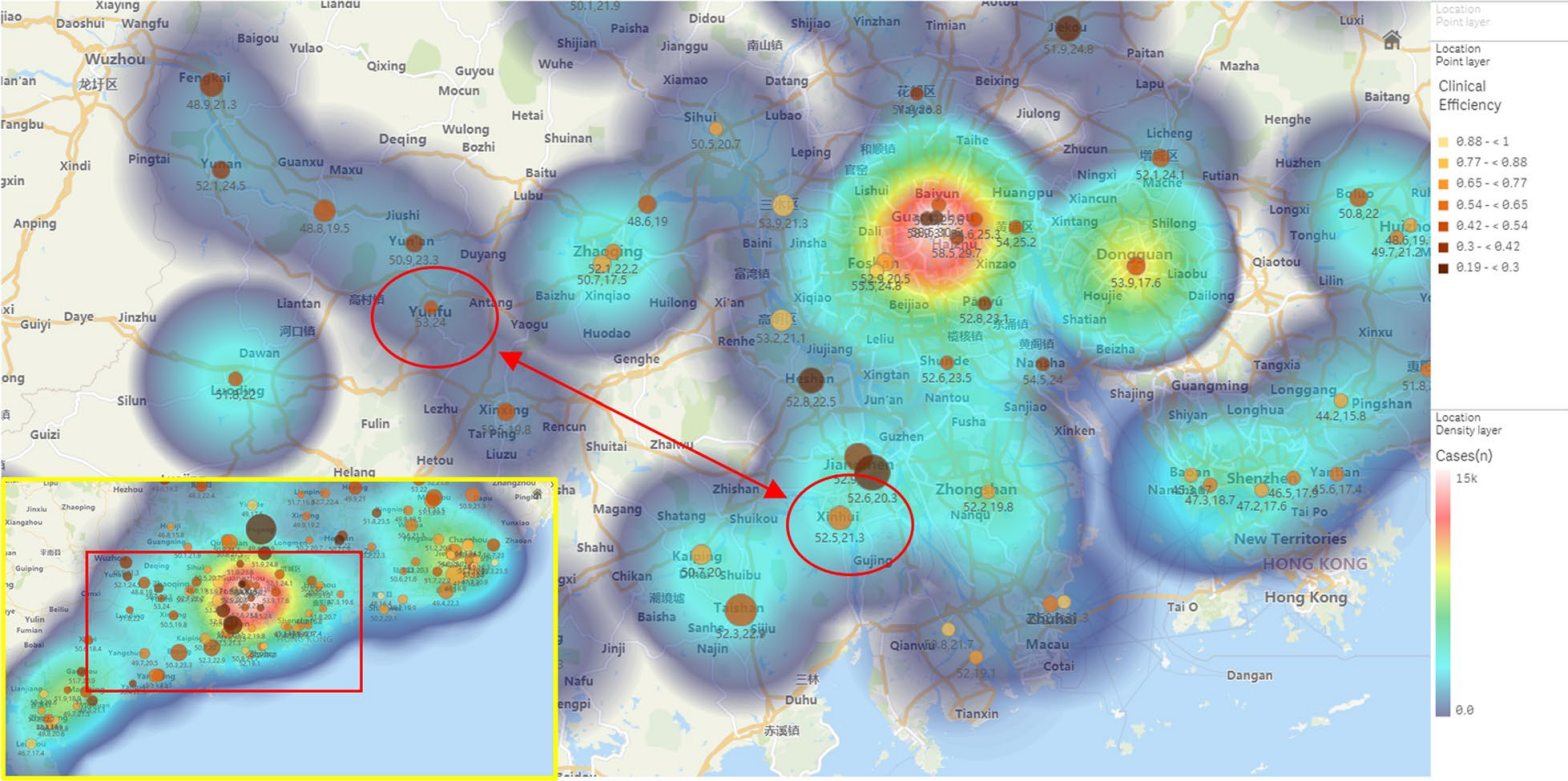
Disease treatment efficiency and risk distributions of Guangdong province for schizophrenia risk early warning. In the map, the higher the population risk score, the larger in node size. The worse clinical effect is, the darker red in node color. The greater crowd density, the brighter blue in cloud color

The autoAHP framework also can improve the current risk warning. By using heat map, the distributions of disease treatment efficiency and risk of population are visualized in Fig. 5. The size and color of the nodes denote clinical efficacy and population risk scores respectively. Through the visualization, clinicians or decision makers find the areas with similar disease risks more accurately. We combine the results of the analysis and related international research to promote the government to carry out real-world research on the application of second-generation anti-schizophrenia long-acting injections to nearly 20,000 patients in Yunfu and Xinhui cities of Guangdong Province, China. Through the verification of clinical empirical research, this framework is able to facilitate the evaluation of the risks and benefits of patient interventions.

## Conclusion

Disease risk assessment, high-risk patients screening, and clinical treatment prediction are critical and practical for schizophrenia patient care. This paper proposes an AutoAHP framework through the combination with automated feature screening, mixed-effect model of APC, logistic regression, Cox model and other single factor, and multi-factor analysis. The framework effectively sorts out disease risk factors, analyzes the factors that affect the treatment of schizophrenia patients, and evaluates multiple indicators to provide auxiliary decision support for chronic disease management.

## Supplementary Information

**Additional file 1: Table S1.** Feature statistics. **Table S2.** The details of final features in AutoAHP framework

## Data Availability

The data analyzed in this study are from Guangdong Mental Health center with approval. The data is unavailable without necessary permission from the center.

## Abbreviations

APC: Age, period and course of disease
AHP: Analytic hierarchy process
IG: Information gain
FCBF: Fast binary feature selection
