# Supplementary material for: Disease risk analysis for schizophrenia patients by an automatic AHP framework
Source: BMC Med Inform Decis Mak. 2022 Jan 11;21(Suppl 9):375. doi: 10.1186/s12911-022-01749-1 (PMC8750858; doi:10.1186/s12911-022-01749-1)
Supplement: Supplementary file 1 — Additional file 1: Table S1. Feature statistics. Table S2. The details of final features in AutoAHP framework [file 12911_2022_1749_MOESM1_ESM.docx]

**Appendix**

**Table 1: Feature statistics**

| Features | Distribution | Center | Disperison | Min | Max | Missing number |
| --- | --- | --- | --- | --- | --- | --- |
| Adverse reactions times | 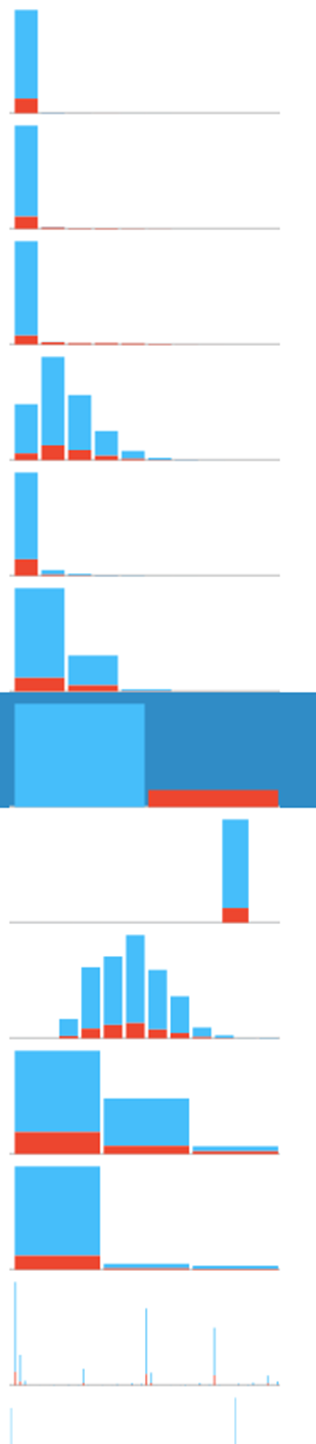 | 0.403 | 6.387 | 0 | 130 | 8 |
| Referral times |  | 1.407 | 4.779 | 0 | 126 | 283 |
| Suggest Referral times |  | 0.984 | 3.832 | 0 | 42 | 5100 |
| Duration days |  | 5880.544 | 0.675 | 0 | 32864 | 0 |
| Hospitalization times |  | 1.383 | 0.568 | 1 | 12 | 0 |
| Drug combination num |  | 1.284 | 0.377 | 1 | 5 | 0 |
| Risk event occurence（Class） |  | 0 | 0.409 | 0 | 1 | 0 |
| Course of disease rating |  | 7 | 0.01 | 0 | 8 | 0 |
| Age rating |  | 44.0-53.5 | 1.813 | 0 | 11 | 0 |
| Social function |  | 1 | 0.805 | 0 | 2 | 920445 |
| Compliance |  | 1 | 0.354 | 0 | 2 | 103 |
| Auxiliary drug combination |  | No | 2.025 | 0 | 54 | 0 |
| Drug combination | 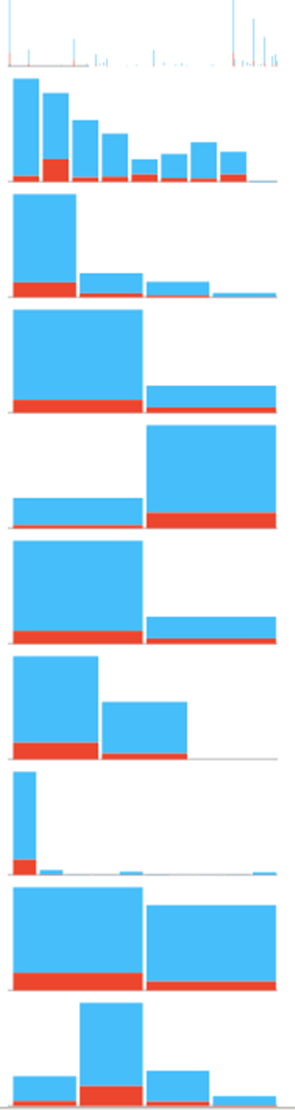 | 2 | 2.714 | 0 | 255 | 0 |
| Region |  | Pearl River Delta | 1.957 | 0 | 8 | 0 |
| Education level |  | Elementary education | 0.887 | 0 | 3 | 77085 |
| Poverty |  | 0 | 0.512 | 0 | 1 | 1573 |
| Family guardianship subsidy |  | 1 | 0.537 | 0 | 1 | 1573 |
| Targeted poverty alleviation |  | 0 | 0.512 | 0 | 1 | 1573 |
| Economic status |  | 1 | 0.653 | 0 | 2 | 0 |
| Diagnostic type |  | F20 | 0.498 | 0 | 9 | 0 |
| Gender |  | 1 | 0.689 | 0 | 1 | 0 |
| Disability rating |  | 2 | 1.096 | 0 | 3 | 750870 |


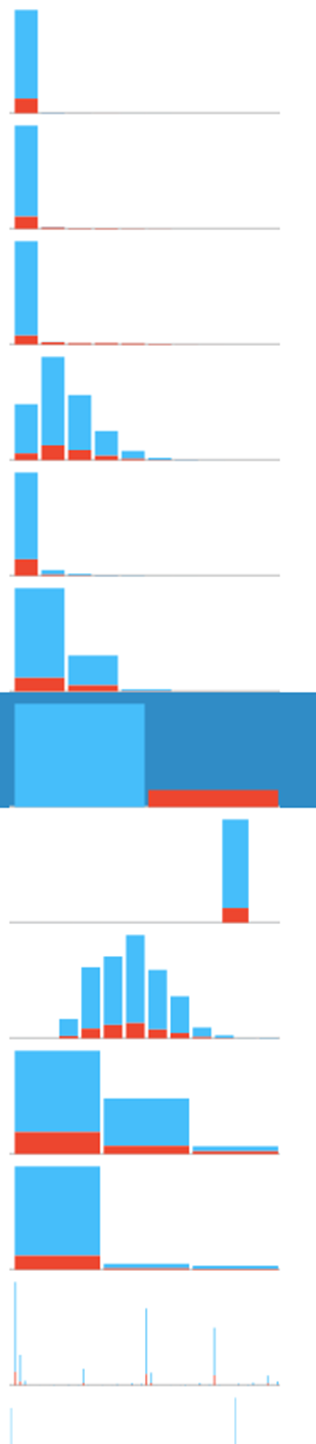

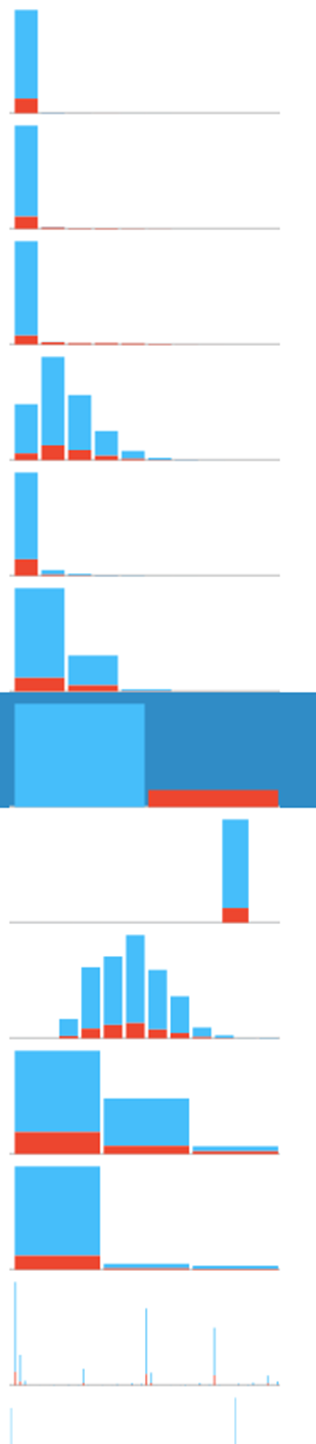


**Table 2: The details of final features in AutoAHP framework**

| Feature | Segment-based thresholds |  |  | Weight |
| --- | --- | --- | --- | --- |
| Referral times | [{'n': '0.0', 'v': 0.0}, {'n': '14.3', 'v': 0.341}, {'n': '28.6', 'v': 0.681}, {'n': '42.0', 'v': 1.0}] | | | 7.374 |
| Suggest Referral times | [{'n': '0.0~', 'v': 0.111}, {'n': '14.0~', 'v': 0.222}, {'n': '28.0~', 'v': 0.333}, {'n': '42.0~', 'v': 0.444}, {'n': '56.0~', 'v': 0.556}, {'n': '70.0~', 'v': 0.667}, {'n': '84.0~', 'v': 0.778}, {'n': '98.0~', 'v': 0.889}, {'n': '112.0~', 'v': 1.0}] | | | 4.754 |
| Adverse reactions times | [{'n': '0.0~', 'v': 0.111}, {'n': '14.4~', 'v': 0.222}, {'n': '28.9~', 'v': 0.333}, {'n': '43.3~', 'v': 0.444}, {'n': '57.8~', 'v': 0.556}, {'n': '72.2~', 'v': 0.667}, {'n': '86.7~', 'v': 0.778}, {'n': '101.1~', 'v': 0.889}, {'n': '115.6~', 'v': 1.0}] | | | 2.226 |
| Region | [{'n': 'Pearl River Delta', 'v': 0.0}, {'n': 'Southern Coastal area of Guangdong', 'v': 0.101}, {'n': 'Mountainous areas in western Guangdong', 'v': 0.138}, {'n': 'Xinfeng-Heyuan', 'v': 0.18}, {'n': 'Western coastal', 'v': 0.238}, {'n': 'Shaoguan, northern Guangdong', 'v': 0.26}, {'n': 'other', 'v': 0.346}, {'n': 'Remote mountainous areas in northern Guangdong', 'v': 0.532}, {'n': 'Chaoshan, Meizhou', 'v': 1.0}] | | | 1.362 |
| Poverty | [{'n': '0', 'v': 0.0}, {'n': '1', 'v': 1.0}] | | | 1.207 |
| Targeted poverty alleviation | [{'n': '0', 'v': 0.0}, {'n': '1', 'v': 1.0}] | | | 1.207 |
| Compliance | [{'n': '1.0', 'v': 0.0}, {'n': '3.0', 'v': 0.965}, {'n': '2.0', 'v': 1.0}] | | | 1.197 |
| Education level | [{'n': 'Elementary education', 'v': 0.0}, {'n': 'High school education', 'v': 0.809}, {'n': 'Higher Education', 'v': 0.881}, {'n': 'Illiterate and semi illiterate', 'v': 1.0}] | | | 1.069 |
| Diagnostic type | [{'n': 'F20', 'v': 0.0}, {'n': 'F20.0', 'v': 0.413}, {'n': 'F20.3', 'v': 0.508}, {'n': 'F20.9', 'v': 0.768}, {'n': 'F20.5', 'v': 0.977}, {'n': 'F20.6', 'v': 0.98}, {'n': 'F20.4', 'v': 0.982}, {'n': 'F20.1', 'v': 0.988}, {'n': 'F20.8', 'v': 0.989}, {'n': 'F20.2', 'v': 1.0}] | | | 1.021 |
| Auxiliary drug *combination | [{'n': '①', 'v': 0.0}, {'n': '③', 'v': 0.664}, {'n': '①,③', 'v': 0.671}, {'n': '①,②', 'v': 0.796}, {'n': '④', 'v': 0.883}, {'n': '⑤', 'v': 0.91}, {'n': '①,③,②', 'v': 0.927}, {'n': '①,⑤', 'v': 0.939}, {'n': '③,④', 'v': 0.941}, {'n': '③,⑤', 'v': 0.941}, {'n': '①,④', 'v': 0.944}, {'n': '②,⑤', 'v': 0.945}, {'n': '②,④', 'v': 0.948}, {'n': '①,②,⑤', 'v': 0.951}, {'n': '①,③,⑤', 'v': 0.951}, {'n': '⑤,⑥', 'v': 0.954}, {'n': '①,③,④', 'v': 0.954}, {'n': '③,②,⑤', 'v': 0.955}, {'n': '①,③,⑥', 'v': 0.956}, {'n': '①,③,②,⑤', 'v': 0.958}, {'n': '①,③,②,⑥', 'v': 0.958}, {'n': '③,②', 'v': 0.958}, {'n': '⑤,④', 'v': 0.958}, {'n': '①,③,②,④', 'v': 0.958}, {'n': '①,③,⑤,⑥', 'v': 0.958}, {'n': '①,⑤,④', 'v': 0.959}, {'n': '①,⑥,④', 'v': 0.959}, {'n': '③,⑤,④', 'v': 0.959}, {'n': '③,⑥,④', 'v': 0.96}, {'n': '③,②,⑥,④', 'v': 0.96}, {'n': '②,⑤,⑥', 'v': 0.96}, {'n': '①,③,⑤,④', 'v': 0.96}, {'n': '③,⑤,⑥', 'v': 0.96}, {'n': '②,⑥,④', 'v': 0.96}, {'n': '②,⑤,⑥,④', 'v': 0.96}, {'n': '①,③,②,⑥,④', 'v': 0.96}, {'n': '①,③,⑥,④', 'v': 0.96}, {'n': '①,⑤,⑥', 'v': 0.96}, {'n': '①,②,⑤,④', 'v': 0.96}, {'n': '②,⑤,④', 'v': 0.96}, {'n': '①,②,⑤,⑥', 'v': 0.96}, {'n': '③,②,⑤,④', 'v': 0.96}, {'n': '③,②,⑤,⑥', 'v': 0.96}, {'n': '①,③,②,⑤,⑥', 'v': 0.96}, {'n': '①,②,⑥,④', 'v': 0.961}, {'n': '①,②,⑥', 'v': 0.961}, {'n': '⑥,④', 'v': 0.964}, {'n': '③,②,④', 'v': 0.965}, {'n': '②,⑥', 'v': 0.967}, {'n': '①,②,④', 'v': 0.967}, {'n': '③,②,⑥', 'v': 0.967}, {'n': '⑥', 'v': 0.976}, {'n': '③,⑥', 'v': 0.982}, {'n': '②', 'v': 0.995}, {'n': '①,⑥', 'v': 1.0}] | | | 1.02 |
| Disability rating | [{'n': '3', 'v': 0.0}, {'n': '4', 'v': 0.145}, {'n': '1', 'v': 0.697}, {'n': '2', 'v': 1.0}] | | | 1.016 |
| Family guardianship subsidy | [{'n': '0', 'v': 0.0}, {'n': '1', 'v': 1.0}] | | | 1.015 |
| Age rating | [{'n': '53.5-63.0', 'v': 0.0}, {'n': '25.0-34.5', 'v': 0.149}, {'n': '44.0-53.5', 'v': 0.168}, {'n': '15.5-25.0', 'v': 0.396}, {'n': '63.0-72.5', 'v': 0.439}, {'n': '<15.5', 'v': 0.88}, {'n': '101.0-110.5', 'v': 0.896}, {'n': 'above 110.5', 'v': 0.896}, {'n': '82.0-91.5', 'v': 0.897}, {'n': '72.5-82.0', 'v': 0.907}, {'n': '91.5-101.0', 'v': 0.913}, {'n': '34.5-44.0', 'v': 1.0}] | | | 1 |
| Course of disease rating | [{'n': 'above 80.5', 'v': 0.0}, {'n': '23.5-33.0', 'v': 0.999}, {'n': '4.5-14.0', 'v': 0.999}, {'n': '<4.5', 'v': 0.999}, {'n': '52.0-61.5', 'v': 0.999}, {'n': '14.0-23.5', 'v': 1.0}, {'n': '42.5-52.0', 'v': 1.0}, {'n': '61.5-71.0', 'v': 1.0}, {'n': '33.0-42.5', 'v': 1.0}] | | | 1 |
| *Drug combination | [{'n': '3', 'v': 0.0}, {'n': '2', 'v': 0.054}, {'n': '11', 'v': 0.093}, {'n': '4', 'v': 0.198}, {'n': '7', 'v': 0.208}, {'n': '10', 'v': 0.343}, {'n': '6', 'v': 0.357}, {'n': '14', 'v': 0.413}, {'n': '11,4', 'v': 0.414}, {'n': '11,6', 'v': 0.445}, {'n': '1,11', 'v': 0.446}, {'n': '11,2', 'v': 0.45}, {'n': '3,7', 'v': 0.45}, {'n': '2,4', 'v': 0.45}, {'n': '11,7', 'v': 0.452}, {'n': '2,7', 'v': 0.452}, {'n': '12', 'v': 0.453}, {'n': '11,3', 'v': 0.453}, {'n': '6,7', 'v': 0.454}, {'n': '4,6', 'v': 0.455}, {'n': '10,3', 'v': 0.457}, {'n': '14,3', 'v': 0.457}, {'n': '10,4', 'v': 0.457}, {'n': '1,14', 'v': 0.459}, {'n': '4,7', 'v': 0.459}, {'n': '14,7', 'v': 0.46}, {'n': '10,7', 'v': 0.46}, {'n': '11,14', 'v': 0.46}, {'n': '10,11', 'v': 0.46}, {'n': '3,4', 'v': 0.461}, {'n': '14,4', 'v': 0.461}, {'n': '10,14', 'v': 0.462}, {'n': '1,12', 'v': 0.462}, {'n': '2,3,4', 'v': 0.462}, {'n': '3,6', 'v': 0.462}, {'n': '3,4,6', 'v': 0.462}, {'n': '2,4,6', 'v': 0.462}, {'n': '12,3', 'v': 0.462}, {'n': '1,2,9', 'v': 0.462}, {'n': '11,4,6', 'v': 0.462}, {'n': '11,3,4', 'v': 0.462}, {'n': '1,10,4', 'v': 0.463}, {'n': '1,10,3', 'v': 0.463}, {'n': '12,7', 'v': 0.463}, {'n': '11,9', 'v': 0.463}, {'n': '11,2,4', 'v': 0.463}, {'n': '1,4,7', 'v': 0.463}, {'n': '2,4,7', 'v': 0.463}, {'n': '2,6,7', 'v': 0.463}, {'n': '1,10,11', 'v': 0.463}, {'n': '1,11,14', 'v': 0.463}, {'n': '14,6', 'v': 0.463}, {'n': '12,2', 'v': 0.463}, {'n': '11,6,7', 'v': 0.463}, {'n': '1,6,7', 'v': 0.463}, {'n': '11,3,7', 'v': 0.463}, {'n': '1,14,3', 'v': 0.463}, {'n': '11,3,6', 'v': 0.463}, {'n': '10,11,9', 'v': 0.463}, {'n': '10,2,3', 'v': 0.463}, {'n': '11,2,7', 'v': 0.463}, {'n': '14,3,7', 'v': 0.463}, {'n': '11,14,3', 'v': 0.463}, {'n': '3,6,7', 'v': 0.463}, {'n': '10,9', 'v': 0.463}, {'n': '1,7,9', 'v': 0.463}, {'n': '14,2,3,6', 'v': 0.463}, {'n': '2,3,9', 'v': 0.463}, {'n': '1,11,2,4', 'v': 0.463}, {'n': '11,14,2', 'v': 0.463}, {'n': '1,14,6', 'v': 0.463}, {'n': '1,10,6', 'v': 0.463}, {'n': '1,3,6', 'v': 0.463}, {'n': '10,3,6', 'v': 0.463}, {'n': '14,2,4', 'v': 0.463}, {'n': '14,2,6', 'v': 0.463}, {'n': '1,14,4', 'v': 0.463}, {'n': '10,3,7', 'v': 0.463}, {'n': '2,6', 'v': 0.463}, {'n': '10,3,4', 'v': 0.463}, {'n': '11,14,4', 'v': 0.463}, {'n': '10,11,2', 'v': 0.463}, {'n': '13', 'v': 0.463}, {'n': '6,9', 'v': 0.463}, {'n': '11,12', 'v': 0.463}, {'n': '1,14,7', 'v': 0.463}, {'n': '11,4,9', 'v': 0.463}, {'n': '1,10,14', 'v': 0.463}, {'n': '14,3,6', 'v': 0.463}, {'n': '1,11,4,6', 'v': 0.463}, {'n': '1,2,4,7', 'v': 0.463}, {'n': '14,9', 'v': 0.463}, {'n': '10,14,6', 'v': 0.463}, {'n': '10,11,4', 'v': 0.463}, {'n': '1,2,3,4', 'v': 0.463}, {'n': '1,3,9', 'v': 0.463}, {'n': '10,11,7', 'v': 0.463}, {'n': '1,14,2,6', 'v': 0.463}, {'n': '10,2,3,6', 'v': 0.463}, {'n': '10,4,7', 'v': 0.463}, {'n': '10,3,9', 'v': 0.463}, {'n': '14,2,3', 'v': 0.463}, {'n': '11,14,6', 'v': 0.463}, {'n': '2,6,9', 'v': 0.463}, {'n': '1,3,6,7', 'v': 0.463}, {'n': '1,2,3,7', 'v': 0.463}, {'n': '1,2,4,9', 'v': 0.463}, {'n': '14,6,7', 'v': 0.463}, {'n': '10,11,6', 'v': 0.463}, {'n': '4,7,9', 'v': 0.463}, {'n': '1,2,4,6', 'v': 0.463}, {'n': '10,14,2', 'v': 0.463}, {'n': '11,6,9', 'v': 0.463}, {'n': '1,10,2,4', 'v': 0.463}, {'n': '1,11,14,2', 'v': 0.463}, {'n': '1,2,3,6', 'v': 0.463}, {'n': '14,4,7', 'v': 0.463}, {'n': '11,2,4,9', 'v': 0.463}, {'n': '1,10,2,7', 'v': 0.463}, {'n': '11,14,7', 'v': 0.463}, {'n': '14,4,6', 'v': 0.463}, {'n': '11,2,3,4', 'v': 0.463}, {'n': '2,3,4,7', 'v': 0.463}, {'n': '1,11,2,7', 'v': 0.463}, {'n': '1,11,2,9', 'v': 0.463}, {'n': '12,14', 'v': 0.463}, {'n': '10,2,3,7', 'v': 0.463}, {'n': '1,11,4,9', 'v': 0.463}, {'n': '10,4,6', 'v': 0.463}, {'n': '1,2,6,7', 'v': 0.463}, {'n': '3,7,9', 'v': 0.463}, {'n': '1,10,12', 'v': 0.463}, {'n': '10,6,9', 'v': 0.463}, {'n': '12,6,7', 'v': 0.463}, {'n': '3,4,9', 'v': 0.463}, {'n': '1,11,3,7', 'v': 0.463}, {'n': '11,12,2,3', 'v': 0.463}, {'n': '12,2,3', 'v': 0.463}, {'n': '10,11,14,4', 'v': 0.463}, {'n': '1,3,4,6', 'v': 0.463}, {'n': '12,3,6', 'v': 0.463}, {'n': '1,11,3,4,6', 'v': 0.463}, {'n': '4,6,9', 'v': 0.463}, {'n': '1,10,2,3', 'v': 0.463}, {'n': '11,4,6,7', 'v': 0.463}, {'n': '2,7,9', 'v': 0.463}, {'n': '1,14,9', 'v': 0.463}, {'n': '1,12,7', 'v': 0.463}, {'n': '12,2,6', 'v': 0.463}, {'n': '10,14,7', 'v': 0.463}, {'n': '10,11,2,3', 'v': 0.463}, {'n': '11,2,4,7', 'v': 0.463}, {'n': '1,10,11,7', 'v': 0.463}, {'n': '14,2,9', 'v': 0.463}, {'n': '7,9', 'v': 0.463}, {'n': '11,2,3,7', 'v': 0.463}, {'n': '1,6,7,9', 'v': 0.463}, {'n': '12,4,6,7', 'v': 0.463}, {'n': '12,3,9', 'v': 0.463}, {'n': '1,11,3,9', 'v': 0.463}, {'n': '10,7,9', 'v': 0.463}, {'n': '10,11,3,6', 'v': 0.463}, {'n': '2,3,4,6', 'v': 0.463}, {'n': '10,11,2,6', 'v': 0.463}, {'n': '1,12,4', 'v': 0.463}, {'n': '12,14,3', 'v': 0.463}, {'n': '1,11,3,6', 'v': 0.463}, {'n': '12,3,7', 'v': 0.463}, {'n': '1,10,6,7', 'v': 0.463}, {'n': '13,3', 'v': 0.463}, {'n': '1,11,14,4', 'v': 0.463}, {'n': '1,11,14,3', 'v': 0.463}, {'n': '2,3,6,7', 'v': 0.463}, {'n': '11,14,2,6', 'v': 0.463}, {'n': '12,2,7', 'v': 0.463}, {'n': '1,14,2,4', 'v': 0.463}, {'n': '1,14,4,7', 'v': 0.463}, {'n': '10,4,9', 'v': 0.463}, {'n': '10,6,7', 'v': 0.463}, {'n': '1,11,3,4,7', 'v': 0.463}, {'n': '11,3,6,7', 'v': 0.463}, {'n': '1,10,11,14', 'v': 0.463}, {'n': '1,10,11,6', 'v': 0.463}, {'n': '3,6,9', 'v': 0.463}, {'n': '11,2,3,9', 'v': 0.463}, {'n': '10,2,3,4,9', 'v': 0.463}, {'n': '10,2,4,7', 'v': 0.463}, {'n': '1,11,6,9', 'v': 0.463}, {'n': '3,4,7', 'v': 0.463}, {'n': '1,12,2', 'v': 0.463}, {'n': '1,11,2', 'v': 0.463}, {'n': '11,7,9', 'v': 0.463}, {'n': '10,14,3', 'v': 0.463}, {'n': '1,6,9', 'v': 0.463}, {'n': '14,3,4', 'v': 0.463}, {'n': '12,4', 'v': 0.463}, {'n': '1,11,9', 'v': 0.463}, {'n': '10,11,3', 'v': 0.463}, {'n': '11,3,9', 'v': 0.463}, {'n': '1,11,12', 'v': 0.463}, {'n': '1,3,4,7', 'v': 0.463}, {'n': '14,2,3,6,7', 'v': 0.463}, {'n': '14,2,7', 'v': 0.464}, {'n': '1,11,2,3', 'v': 0.464}, {'n': '11,4,7', 'v': 0.464}, {'n': '10,2,7', 'v': 0.464}, {'n': '1,11,3,4', 'v': 0.464}, {'n': '1,10,2,6', 'v': 0.464}, {'n': '1,11,6,7', 'v': 0.464}, {'n': '10,3,4,6', 'v': 0.464}, {'n': '4,6,7', 'v': 0.464}, {'n': '2,4,9', 'v': 0.464}, {'n': '1,14,2', 'v': 0.464}, {'n': '14,2', 'v': 0.464}, {'n': '1,12,3', 'v': 0.464}, {'n': '11,2,6', 'v': 0.464}, {'n': '11,2,3', 'v': 0.464}, {'n': '2,3,6', 'v': 0.464}, {'n': '1,11,2,6', 'v': 0.464}, {'n': '1,2,4', 'v': 0.464}, {'n': '10,12,3', 'v': 0.464}, {'n': '1,14,3,7', 'v': 0.464}, {'n': '10,12', 'v': 0.464}, {'n': '1,11,7', 'v': 0.464}, {'n': '3,9', 'v': 0.465}, {'n': '1,3,7', 'v': 0.465}, {'n': '10,2,4', 'v': 0.465}, {'n': '10,2,6', 'v': 0.465}, {'n': '1,11,3', 'v': 0.465}, {'n': '10,6', 'v': 0.465}, {'n': '1,7', 'v': 0.465}, {'n': '4,9', 'v': 0.465}, {'n': '1,11,4', 'v': 0.465}, {'n': '1,11,6', 'v': 0.466}, {'n': '2,3,7', 'v': 0.466}, {'n': '10,2', 'v': 0.466}, {'n': '11,2,9', 'v': 0.466}, {'n': '1,10,2', 'v': 0.467}, {'n': '1,10,7', 'v': 0.467}, {'n': '1,4,9', 'v': 0.467}, {'n': '2,9', 'v': 0.467}, {'n': '1,3,4', 'v': 0.467}, {'n': '1,4,6', 'v': 0.468}, {'n': '1,2,6', 'v': 0.47}, {'n': '12,6', 'v': 0.471}, {'n': '1,2,3', 'v': 0.472}, {'n': '1,2,7', 'v': 0.474}, {'n': '1,9', 'v': 0.476}, {'n': '1,10', 'v': 0.478}, {'n': '1,3', 'v': 0.481}, {'n': '1,6', 'v': 0.487}, {'n': '2,3', 'v': 0.495}, {'n': '9', 'v': 0.504}, {'n': '1', 'v': 0.523}, {'n': '1,4', 'v': 0.548}, {'n': '1,2', 'v': 1.0}] | | | 0.996 |
| Social function | [{'n': '2.0', 'v': 0.0}, {'n': '3.0', 'v': 0.248}, {'n': '1.0', 'v': 1.0}] | | | 0.916 |
| Economic status | [{'n': '2', 'v': 0.0}, {'n': '3', 'v': 0.966}, {'n': '1', 'v': 1.0}] | | | 0.881 |
| Gender | [{'n': '2', 'v': 0.0}, {'n': '1', 'v': 1.0}] | | | 0.824 |
| Duration days | [{'n': '32864.0~', 'v': 0.111}, {'n': '29065.0~', 'v': 0.222}, {'n': '25266.0~', 'v': 0.333}, {'n': '21467.0~', 'v': 0.444}, {'n': '17668.0~', 'v': 0.556}, {'n': '13869.0~', 'v': 0.667}, {'n': '10070.0~', 'v': 0.778}, {'n': '6271.0~', 'v': 0.889}, {'n': '2472.0~', 'v': 1.0}] | | | 0.773 |
| drug combination num | [{'n': '5.0~', 'v': 0.2}, {'n': '4.0~', 'v': 0.4}, {'n': '3.0~', 'v': 0.6}, {'n': '2.0~', 'v': 0.8}, {'n': '1.0~', 'v': 1.0}] | | | 0.763 |
| Hospitalization times | [{'n': '12.0~', 'v': 0.111}, {'n': '10.6~', 'v': 0.222}, {'n': '9.2~', 'v': 0.333}, {'n': '7.9~', 'v': 0.444}, {'n': '6.5~', 'v': 0.556}, {'n': '5.1~', 'v': 0.667}, {'n': '3.8~', 'v': 0.778}, {'n': '2.4~', 'v': 0.889}, {'n': '1.0~', 'v': 1.0}] | | | 0.218 |

***Drug combination**

1: Clozapine

2: Risperidone

3: Olanzapine

4: Perphenazine

5: Chlorpromazine

6: Quetiapine

7: Aripiprazole

8: Lithium carbonate

9: Pentafluridol

10: Amisulpride

11: Other

***Auxiliary drug combination**

①: Benzhexol (e.g. Benzhexol hydrochloride)

②: Valproate (e.g. Valproate Sodium)

③: Sedative-Hypnotic drugs (e.g. Diazepam)

④: Neuroprotective drugs (e.g. Piracetam)

⑤: Hepatoprotective drugs (e.g. Glucuronolactone)

⑥: Chinese patent medicine (e.g. Seven Leaves Spirit Calmness Tablet)
